# PROTOCOL: Effectiveness of interventions for improving educational outcomes for people with disabilities in low‐ and middle‐income countries: A systematic review

**DOI:** 10.1002/cl2.1197

**Published:** 2021-10-23

**Authors:** Xanthe Hunt, Ashrita Saran, Howard White, Hannah Kuper

**Affiliations:** ^1^ Stellenbosch University Cape Town South Africa; ^2^ Campbell Collaboration Delhi India; ^3^ International Centre for Evidence on Disability, London School of Hygiene & Tropical Medicine London UK

## Abstract

The objectives of this review are to answer the following research questions: (1) What is the nature of the interventions used to support education for people with disabilities? (2) What is the size and quality of the evidence base of the effectiveness of interventions to improve educational outcomes for people with disabilities in low‐ and middle‐income countries (LMICs)? (3) What works to improve educational outcomes for people with disabilities in LMICs? (4) Which interventions appear most effective for different types of disability? (5) What are the barriers and facilitators to improving of educational outcomes for people with disabilities? Is there evidence of cumulative effects—that certain interventions are effective when done in combination with others, but are less or ineffective when done alone?

## 1 BACKGROUND

### 1.1 The problem, condition or issue

People with disabilities are less likely to be enroled in school or to progress as well as their peers without disabilities. Due in part to these inequalities in educational access and attainment, people with disabilities are also less likely to achieve employment (Department of Economic and Social Affairs, n.d.), or earn as much if they are employed (Equality and Human Rights Commission, 2017), as people without disabilities.

Despite the lack of comparable data on education for people with disabilities, recent reports (UNESCO, 2018; World Bank Group, 2019) showed that people with disabilities were consistently falling behind in educational outcomes compared to their peers without disabilities, whether measured in terms of school enrolment, school completion, mean years of schooling, or literacy levels. For instance, UNESCO's 2020 Global Education Monitoring Report (UNESCO, 2020) noted that children with disabilities make up 15% of the out‐of‐school population, and that individuals with a sensory, physical or intellectual disability are two‐and‐a‐half times more likely than nondisabled individual to have never been in school (UNESCO, 2020).

Evidence suggests that the gap in educational attainment for people with and without disabilities is greatest in LMIC. In a 2014 study, children with disabilities were found to be 5–10 times more likely to be excluded from school than children without disabilities, and children with learning or communication impairments were consistently among the least likely to attend school, particularly in Africa (Kuper et al., 2014). This finding has been supported by subsequent analyses; in 2018, a study by Mizunoya et al. (2018) showed that the disability gap in school attendance was statistically significant in all 15 LMIC the authors examined. In these settings, living with a disability reduced the probability of being in school by a median 30.9% (Mizunoya et al., 2018).

Importantly, the study of Mizunoya et al. (2018) indicated that neither individual nor socioeconomic and household characteristics explained the disability gap. This seems to suggest that there is something in the environment of education, for instance in the way schools are structured and functioning, the way learning happens, the way teachers and peers interact with children with disabilities, and other factors not captured by demography, which is keeping children with disabilities out of school, and as such, prohibiting them from achieving positive educational outcomes.

The research from LMIC evidences similar exclusion and inequalities. School attendance and social integration among children with epilepsy in one study in India was found to be lower than among children without epilepsy (Pal et al., 2002), and evidence from Uganda suggests that barriers in the built environment in schools hinder inclusion (Wapling, 2016). Large class sizes (Hove, 2014; Wapling, 2016) and poor attitudes to educating children with disabilities by mainstream school educators (De Boer et al., 2011) are also reported to hinder educational success among children with disabilities in LMIC.

#### 1.1.1 Inclusive education

An important issue which must be flagged when considering disability and education, is the debate around “mainstreaming” or inclusive education, versus “special‐needs” or segregated education. Historically, where people with disabilities were granted access to education, that education mostly happened in so‐called “special” schools ‐ segregated learning environments where only children with disabilities were admitted, and where they would engage in learning separately from children without disabilities.

In the past two decades, there has been a significant shift in this status quo, with a movement from segregated to inclusive education, the latter being learning environments where children with disabilities and children without disabilities are educated together. The right to inclusive education was initially noted in the Salamanca Statement and Framework for Action (UNESCO, 1994). However, it was the United Nations Convention on the Rights of Persons with Disabilities (UNCRPD) (UN General Assembly, 2007) which established inclusive education as a legal right, mandating countries to support its achievement.

Inclusion in the school context refers to the process of bringing children, with or without special education needs, together in the same premises and under the same conditions (Ghergut, 2012). It requires that learning environments which previously catered to relatively homogenous groups of students who learned in similar ways, to adapt and resource the full participation of all pupils, regardless of ability (Ghergut, 2012; Stainback et al., 1998; Stubbs, 2008). It implies contexts beyond school, and if seen to fruition, would see people with disabilities participate in learning that begins at birth and is lifelong, and includes learning in the home, the community, and in formal, informal and nonformal situations (Stubbs, 2008).

Inclusive education, in light of the UNCRPD, is a key tenet of education and/or disability policy in a number of countries (Lindsay, 2007). Yet, the ideal of inclusive education in relation to disability is not without its limitations and complexities. In LMIC and poorly resourced contexts, in particular, lack of experienced teachers and teaching aides in classrooms, high child to teacher ratios, and poor financing for inclusion can result in people with disabilities being “housed” in a mainstream school, but not truly experiencing or benefitting from inclusion in any meaningful way (Wapling, 2016). Even in a well‐resourced settings, inclusive education is a human‐resource intensive undertaking, as teachers must address individual academic needs based on ability if the ideals of inclusion are to be truly achieved.

Further, while special education has long been criticised as segregationist and discriminatory (Lipsky & Gartner, 1987), children with certain types of impairments may benefit from separate instruction where the environment and educators cater to their specific learning needs, and there are issues with social integration, communication, and friendship for children who are deaf in mainstream schools (Wolters et al., 2011). This, as well as the slow pace of transformation to inclusive education in many countries, means that it is important to consider both inclusive and “special school” settings to fully account for the state of education for people with disabilities.

In the context of a systematic review, inclusive education is a thorny issue. First, this is because definitions of what passes as inclusive education differ widely. So‐called inclusive environments range from settings where there simply are not specialised services for children with disabilities and so the assumption is that they are being absorbed into mainstream classrooms, to well‐resourced, integrated classrooms in which children with disabilities participate fully in learning activities with children without disabilities and are provided the supports necessary to participate fully, where such a necessary. Second, where inclusive education is implemented, the models for doing so are numerous, and range from integrating children with disabilities into mainstream schools without additional supports, to including them in mainstream schools through a range of additional supports and accommodations (including, for instance, teaching aides and occupational therapists, specialist training on disability for all teachers etc.). These issues of definition mean that it can be hard for a systematic review to “compare apples with apples”; that is, to draw meaningful connections and comparisons between different interventions calling themselves inclusive education. In this review, we will define inclusive education broadly, according the UNICEF definition:

Inclusive education means all children in the same classrooms, in the same schools. It means real learning opportunities for groups who have traditionally been excluded.

**Figure 1:**
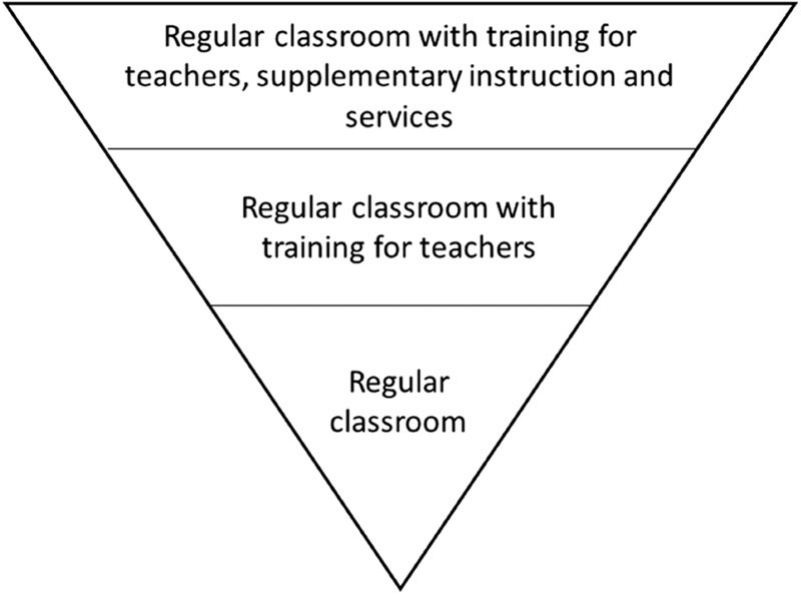
Inverted pyramid of inclusive education intensity

However, in our analysis and synthesis, we will be mindful of the likely heterogeneity within these interventions and take steps to highlight the provisions for inclusion mentioned in each case. For instance, where an inclusive education intervention mentions provision of additional human resources for inclusion (such as teaching aides), training on inclusive education and disability for mainstream teachers, inclusion of specialist staff in the school to support children with disabilities (such as occupational therapists, physiotherapists etc.), and any other measures aimed at moving inclusive education beyond simply moving children with disabilities into mainstream schools, these will be noted, and used to organise inclusive education interventions on a continuum from lowest to highest intensity (see Figure 1, below, for an example of this thinking).

#### 1.1.2 The significance of this review

Exclusion of people with disabilities from mainstream education, and low rates of participation in education of any kind, are important issues. First, people with disabilities have a fundamental right to education. The UNCRPD recognise the right of persons with disabilities to education and calls on states to facilitate their full and equal participation in education, as does the United Nations Convention on Rights of the Child. Furthermore, this exclusion is a development issue as the Sustainable Development Goals call for quality education for all, and includes a target related to the elimination of inequalities in access to education for people with disabilities. Additionally, there are multiple benefits to the inclusion of children with disabilities in schooling, in terms of social participation and improving their future employment prospects. Educational inclusion therefore creates positive outcomes for people with disabilities, both financial and nonfinancial. There are also numerous benefits to including people with disabilities in lifelong learning—education beyond the school years, including nonformal education, and life skills education. Opportunities before school—including early childcare and education (ECCE)—are equally important for all individuals to support the optimisation of development.

To improve educational outcomes for people with disabilities, barriers to inclusion need to be addressed, both in terms of school attendance, experience in school and educational outcomes. These barriers operate at the level of the system (e.g., lack of policy), school (e.g., lack of accessible infrastructure or skilled teachers), and the family/child (e.g., poor health), as highlighted in UNESCO's 2020 Global Education Monitoring Report (UNESCO, 2020). The report notes, for instance, that policy and legislative barriers are prevalent as laws in 25% of countries (but over 40% in Asia and in Latin America and the Caribbean) make provisions for education in separate settings, 10% for integration and only 17% for inclusion of children with disabilities in mainstream schools (UNESCO, 2020).

In response to these circumstances, education is a core component in the World Health Organization's (WHO's) Community Based Rehabilitation (CBR) programme, which is a comprehensive and multisectoral strategy to equalise opportunities and include people with disabilities in all aspects of community life. The guidance document on CBR in education notes the global need to expand and improve the quality, availability, accessibility, and equitability of education for children with disabilities. The CBR programme also has an emphasis on early education, lifelong learning and nonformal education for people with disabilities.

Although these international directives place obligations on states to respect, protect, and fulfil the right to education of people with disabilities, evidence on which interventions are effective to achieve the goals they outline, have not been established. Indeed, past evidence syntheses on the topic of education and disability in LMIC have highlighted that very little literature has examined educational outcomes, comparing disabled and nondisabled peers (Wapling, 2016). Furthermore, a majority of studies have focussed on special school populations and addressed epidemiological questions rather than ones of attendance of attainment (Maulik & Darmstadt, 2007). Consequently, there is a real need to evaluate interventions in the realm of disability and education, to determine “what works” to produce educational inclusion and good educational outcomes for people with disabilities.

#### 1.1.3 A note on defining education

Many low‐ and middle‐income countries (LMICs)—the settings in which this review is interested—are non‐Western and, often, postcolonial settings. This raises an important issue for this review to address, which is, “What do we mean by education?” In many LMICs, low status has historically and is still accorded to indigenous knowledge in comparison to institutional knowledge (for instance, low status may be accorded to the intergenerational passing down of knowledge about which land is arable and which not, and high status accorded to a University degree in agriculture). The systematic review format privileges Western positivist thought, and—while we are willing to include studies which explore indigenous knowledge transfer in the context of disability—we are unlikely to find it by examining published, written literature. As such, the authors must note that the types of education and educational outcomes likely to be privileged in this type of inquiry include those delivered through formal institutions of learning like schools, universities, and vocational training centres, as opposed to other, less quantifiable forms of knowledge transfer.

### 1.2 The intervention

The interventions which will be considered in this review are those that improve educational outcomes for people with disabilities, whether delivered in specialist or inclusive education settings. Such interventions involve a wide range of initiatives, from those focussed at the individual level—such as teaching assistance to make mainstream classes more accessible to children with specific learning needs—to those which address policy or advocacy.

There are existing frameworks for considering how best to support the education and educational inclusion of people with disabilities given the importance of education for a vast number of social, environmental, economic and human capital development goals (Adedeji & Campbell, 2013; Nazar et al., 2018; Vladimirova & Le Blanc, 2016; Walid & Luetz, 2018). We consider the scope of education in line with the WHO's CBR Guidelines (WHO, 2010). CBR promotes the equalisation of opportunities between disabled people and people without disabilities, and strives for the widespread inclusion of people with disabilities in all spheres of life. The WHO CBR Guidelines has “education” as one of its five pillars (WHO, 2010). As such, the Guidelines see education interventions as key to their multisectoral approach (WHO, 2010).

**Figure 2:**
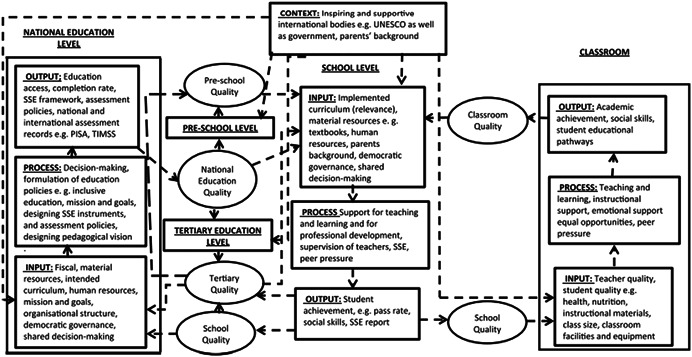
Garira's (2020) unified framework for quality education

Garira (2020) proposes a unified conceptual framework for quality education in schools. This framework (see Figure 2) highlights the conditions required for quality education at various levels. Taking a systemic approach to quality education, the unified framework is based on an input, process, output approach, where inputs, processes, and outputs can be specified at the national, preschool, tertiary, and school levels (Garira, 2020).

As Love and Horn (2021) note, high‐quality inclusive education depends on people with disabilities being included into already high‐quality learning environments, and—as such—global education quality is a necessary foundation for high‐quality inclusive education. This means that a framework such as Garira's (2020) can useful inform the conceptualisation of quality inclusive education. Nonetheless, there are certain preconditions for quality inclusive education which can be useful highlighted. They draw on the Division for Early Childhood and National Association for the Education of Young Childhood (2009) conceptualisation of inclusive education as (a) access to a wide variety of learning opportunities, (b) individualised modifications that facilitate participation with adults and peers, and (c) systems‐level supports that undergird classroom efforts (e.g., professional development). Love and Horn's (2021) conceptual framework for quality, then, focuses on access to quality opportunities, supports which are adequate and enable meaningful participation, and structural changes to facilitate inclusion.

For the purpose of this review, interventions will be organised around the education pillar of the CBR matrix but expanded based on pilot coding of relevant papers. However, the intervention components and levels of intervention are reflective of the broader literature on quality education and quality inclusive education, including those frameworks noted above. The interventions of interest include:

### 1.3 How the intervention might work

Interventions which aim to improve educational outcomes for people with disabilities have a variety of foci. They include ensuring that:

- Learning environments, including schools, take in all children, including children with disabilities;
- Learning environments, including schools, are inclusive and welcoming and that educators and peers are trained and supported to create an inclusive space for learning by children with disabilities;
- Learning environments, including schools, have adequate infrastructure to be accessible to people with disabilities and provide accessible learning materials;
- People with disabilities are involved in education as role‐models, educators, policymakers, decision‐makers and contributors;
- The home environments of people with disabilities encourage and support learning;
- Communities are aware that people with disabilities can learn;
- Multisectoral collaboration between the health, education, social and other sectors is established and maintained;
- Rehabilitation and health services, and assistive technologies, are available to learners with disabilities to ensure that they can fully and meaningfully participate in and benefit from, educational opportunities; and
- National policies are comprehensive and facilitate inclusive education (WHO, 2010).

These different categories of intervention can be conceived of in clusters along a causal chain.


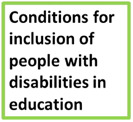


The first set of interventions pertain to (a) addressing the structural forces shaping the context in which education happen, and (b) improving the conditions of a learning environment to better facilitate education for people with disabilities. Structural interventions include those aiming to alleviate poverty, reduce community‐wide stigma against people with disabilities, and/or improve the resources allocated to education at a national or regional level. While many structural interventions may not measure educational outcomes, where they do, these interventions will be eligible for inclusion in this review, as altering the context in which education happens for people with disabilities, in a way which improves educational outcomes, is an educational intervention. In respect of the immediate conditions in which learning happen, modifications to the school social environment and levels of social inclusion for people with disabilities, accessibility of the built environment and learning materials, educational services development and implementation and resourcing of inclusive education and anti‐bullying policies, all contribute to conditions conducive to educational participation by people with disabilities. At this level of intervention, one would also expect to see that rehabilitation and health services, and assistive technologies, are available to learners with disabilities to ensure that they are able to fully and meaningfully participate in and benefit from, educational opportunities.


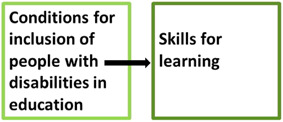


The second cluster of interventions which may improve educational outcomes among people with disabilities are those which aim to equip people with disabilities with the skills necessary to engage in learning. These interventions include a broad range of initiatives in the formal and nonformal sectors, delivered to individuals of all ages, which aim to equip disabled learners with skills for formal learning (for instance, learning in schools), school readiness programming, and those which are more broadly focussed on developing individuals with disabilities' with life skills.


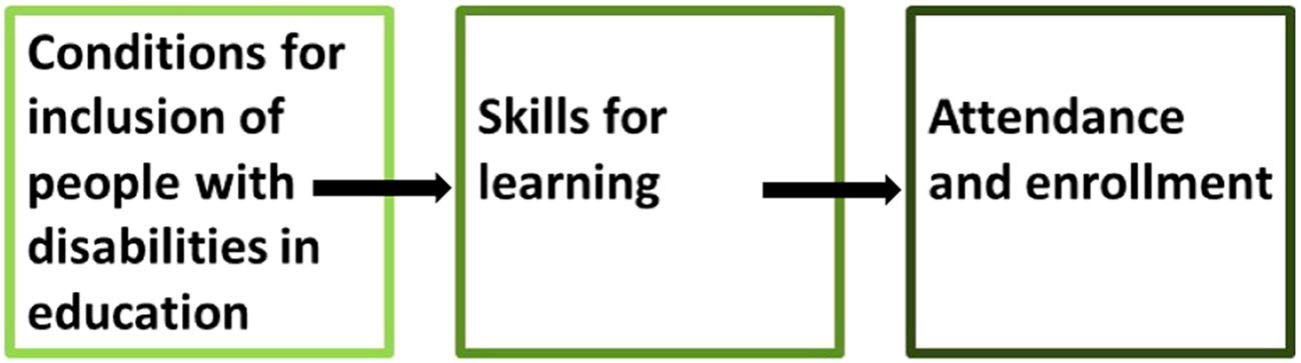


Once learning environments are made accessible, and people with disabilities are equipped with the tools necessary to engage in further learning, interventions aimed at improving attendance and enrolment of people with disabilities in a variety of forms of learning, become important. Programming focused on increasing levels of formal enrolment and nonformal enrolment/participation in nonformal education, as well as those focussed on improving inclusion of disabled people in inclusive/mainstream education settings, form part of this cluster. Such interventions seek to improve school completion and attendance among people with disabilities, given that successful educational attainment is largely predicated on educational participation.


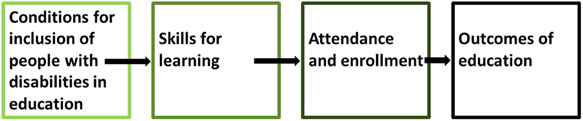


The final cluster of interventions are those which have to do with educational outcomes. Interventions which aim to equip people with disabilities with qualifications or improve through‐put rates of people with disabilities at various stages of education, those seeking to improve the education‐related quality of life of disabled students, and interventions aimed at supporting transitions between different levels of education.

At each phase of life, specific programmes at each of these levels of intervention can ensure that people with disabilities are included in mainstream education, have access to specialised educational services where required/desired, are able to learn to the best of their ability, in an accessible environment, and attain the best possible educational outcomes that they can. Additionally, throughout these stages, a supportive legal/policy environment is important to encourage inclusive education.

For instance, in early childhood (birth to the age of 8 years), early childhood care and education programmes can put in place interventions to improve access to early childhood education, early intervention and preschool/kindergarten for children with disabilities. These interventions are extremely important because early childhood is a period of rapid brain development, and appropriate engagement with children during this phase is imperative to ensure that they develop to their full potential. Interventions during this phase for children with disabilities can ensure that the physical, social, language and cognitive skills of all children are developed to their maximum potential, by:

- Making formal and nonformal early childhood education is welcoming and inclusive of all children;
- Equipping adults in the home and community to appropriately support and include children with disabilities in educational activities;
- Providing opportunities for children with and without disabilities to play together, accept the differences between them and help each other;
- Providing opportunities for stimulation and responsive caregiving so that the impact of impairments is reduced and compensated for; and
- Improving the likelihood that children with disabilities are able to make a smooth transition to primary schooling together with their peers without disability (WHO, 2010).

Primary education, which begins at age 6 or 7 years and continues into the early teen years, is the pathway to higher levels of education. As such, in the context of human development goals, it is extremely important. Interventions during this phase for children with disabilities can help to create a welcoming, inclusive primary education system where all children are able to fulfil their potential, achieve the best possible educational outcomes, and are well positioned to progress to higher forms of education, should they choose/desire to. This can be achieved through:

- Initiatives to mobilise the whole community to develop inclusive primary education;
- Programmes to equip families to support their children's involvement in inclusive primary education;
- Initiatives aimed at improving the quality of inclusive primary education;
- Interventions aimed at ensuring that appropriate assistive devices, therapies and other necessary assistance are accessible and available to support inclusion;
- Training and education for teachers so that they feel supported and are confident in their abilities to educate children with disabilities;
- The development of curricula, examination and assessment systems, teaching approaches and extracurricular activities which are child‐focused and inclusive;
- The development of local and specialist resources for education, including accessible learning materials; and
- Projects which establish and maintain partnerships between relevant stakeholders, with advocacy at all levels, to ensure national policies promote inclusive primary education (WHO, 2010).

Secondary and higher education includes both high school and university academic programmes, as well as a variety of technical/vocational educational opportunities. Interventions to support the inclusion of people with disabilities, and their achievement of the best possible educational outcomes, in these levels of education centre on facilitating inclusion by increasing and improving access, participation and achievement for students with disabilities, and ensuring that learning environments, including teaching and peer environments, are inclusive. Such interventions can achieve these aims through:

- Increasing enrolment, retention and completion in secondary and higher education by students with disabilities;
- Helping students with disabilities to access government grants, scholarships and other sources of funding;
- Ensuring that lobbying groups and campaigns for equal access to education exist and are supported;
- Supporting families and communities to encourage their children, including children with disabilities, to pursue secondary and higher education;
- Making sure that secondary and higher education programmes are accessible and inclusive in terms of environment, teaching methods and materials, curricula, extracurricular activities and assessment and examination systems;
- Promoting learning about diversity and inclusion from the experiences of people with disabilities in secondary schools;
- Providing specialist resources and support to enhance the inclusion of students with disabilities; and
- Supporting transitions between secondary/higher education programmes and into adult life (WHO, 2010).

Finally, nonformal education, sometimes called community education, adult education, and/or lifelong education, refers to educational initiatives which are not “traditional” in the sense of being school or institution based. Such types of education include home‐based learning, government schemes and other local initiatives aimed at improving the knowledge and skills of community members. Interventions to improve access to nonformal education and improve educational outcomes for people with disabilities enroled in nonformal education, may focus on:

- Making sure that nonformal education programmes include people with disabilities and consider their needs during programme planning;
- Involving people with disabilities, family members, disabled people's organisations and parents' associations in decision‐making and implementing nonformal education programmes; and
- Strengthening social cohesion between students with disabilities and nondisabled students (WHO, 2010).

Intervention efforts at each of the above stages of education aim to improve the educational outcomes of people with disabilities through improving access to education, ensuring that educational opportunities are inclusive, making reasonable accommodations to people with disabilities and providing specialised supports where necessary, as well as through a range of other strategies. Target outcomes relate to improving education conditions, access, attendance, and achievement.

### 1.4 Why it is important to do this review

International directives place obligations on states to respect, protect, and fulfil the right to education of people with disabilities, as described above. However, evidence on which interventions are effective to achieve the goals they outline have not been established or comprehensively reviewed.

Several relevant Cochrane and Campbell systematic reviews and protocols exist that are relevant to the topic, but none would address the objectives of this review.

From the Cochrane databases, a review (Pennington et al., 2018) assessed the effectiveness of parent‐mediated communication interventions, for improving the communication skills of preschool children up to 5 years of age who have nonprogressive motor disorders. Also from Cochrane, a review (Cogo‐Moreira et al., 2012) of the evidence concerning music education for improving reading skills in children and adolescents with dyslexia has been conducted. Another Cochrane review has been undertaken on task‐oriented interventions for children with developmental co‐ordination disorder (Miyahara et al., 2017). In all cases, the scope of the reviews is far narrower than our proposed review. In all, the focus is on children with particular conditions (nonprogressive motor disorders, dyslexia and developmental co‐ordination disorder only) and the type of intervention and outcome are narrowly focussed (parent‐mediated interventions, music interventions and task‐oriented interventions only; communication and reading skills only).

Other more topic‐specific rigorous reviews have been conducted and reported in the peer‐reviewed literature (Buysse & Bailey, 1993; Elbaum et al., 1999; Forlin et al., 2013; Gersten et al., 2001; Hudson et al., 2013; Katz & Mirenda, 2002; Paradise et al., 2009; Pierce et al., 2004; Purdie et al., 2002; Reichrath et al., 2010; Ruijs & Peetsma, 2009; Trout et al., 2003; Wapling, 2016).

These include a systematic review of interventions in general education for students with disabilities (Reichrath et al., 2010), but are limited in respect of the:

– Geography of research represented (none are specifically focussed on LMICs);
– Type of review (e.g., nonsystematic, narrative, scoping or reviews of reviews) (reviews type not specified—Forlin et al. (2013); Wapling (2016));
– Impairment type/disabling condition included (emotional and behavioural disorders only—Trout et al. (2003); Pierce et al. (2004); ADHD only—Purdie et al. (2002); Alzheimer's only—Paradise et al. (2009));
– Eligible outcomes included (reading only—Elbaum et al. (1999); Gersten et al. (2001); development and behaviour only—Buysse and Bailey (1993); academic outcomes only—Pierce et al. (2004));
– Other sociodemographic restrictions, such as location or age of the population targeted (both children with and without disabilities—Ruijs and Peetsma (2009); Western contexts only Reichrath et al. (2010));
– Interventions included (inclusive education only—Wapling (Wapling, 2016), Forlin, Chambers (Forlin et al., 2013), Katz and Mirenda (Katz & Mirenda, 2002), Ruijs and Peetsma (Ruijs & Peetsma, 2009); teacher‐mediated interventions only—Pierce, Reid (Pierce et al., 2004)); and/or
– Out of date (Buysse & Bailey, 1993; Elbaum et al., 1999; Trout et al., 2003)

Finally, White et al. (2018) conducted an evidence gap map (EGM)—which can be distinguished from a review in that it is used to identify, map and describe existing evidence of effectiveness studies and highlight gaps in evidence base, and can inform later systematic reviews—on interventions for people with disabilities in LMIC. Their EGM included impact evaluation and systematic reviews assessing the effect of interventions for people with disabilities and their families/carers in LMICs. The EGM included 46 studies related to education outcomes. While we will include all the EGM's included studies in our review, our focus will be narrower (the EGM was broader in scope than our proposed review), and our proposed review will cover an extended publication timeframe.

## 2 OBJECTIVES

The objectives of this review are to answer the following research questions:

1. What is the nature of the interventions used to support education for people with disabilities?
2. What is the size and quality of the evidence base of the effectiveness of interventions to improve educational outcomes for people with disabilities in LMICs?
3. What works to improve educational outcomes for people with disabilities in LMICs?
4. Which interventions appear most effective for different types of disability?
5. What are the barriers and facilitators to the improvement of educational outcomes for people with disabilities?
6. Is there evidence of cumulative effects—that certain interventions are effective when done in combination with others, but are less or un‐effective when done alone?

## 3 METHODS

### 3.1 Criteria for considering studies for this review

#### 3.1.1 Types of studies

In our review, we will include studies on the basis that they are able to detect intervention impact. This includes the following types of studies:

a) Studies where participants are randomly allocated to groups,
b) Studies where a quasi‐random method of participant allocation is used,
c) Studies where participants are not randomly assigned, but are matched on pre‐tests and/or relevant demographic characteristics (using observables, or propensity scores) and/or according to a cut‐off on an ordinal or continuous variable (such as in regression discontinuity study designs),
d) Studies where participants are not randomly assigned, but where statistical methods have been used to control for differences between groups which existed at baseline (for instance, those studies where multiple regression analysis or instrumental variables regression is used),
e) Studies where an interrupted time‐series design is used, and an attempt is made to detect whether the intervention has had an effect which is significantly greater than any underlying trend which would have occurred without intervention over time, using observations at multiple time points before and after the intervention,
f) Studies where a historical control is used, and participants who are receiving an intervention are compared to a similar group from the past who did not receive the same intervention, and
g) Studies where a single‐group before‐and‐after design is employed, and observations are made on a group of individuals before and after an intervention, but with no control group.

Descriptive studies of various designs and methodologies (such as qualitative interview studies, single time point cross‐sectional surveys etc.) will not be included because impact evaluations are better suited to answering the questions about effectiveness such as “What works to improve educational outcomes for people with disabilities in LMICs?”—the purpose of the proposed review.

#### 3.1.2 Types of participants

We will include studies which examine the impact of interventions for the following population: people with disabilities living in LMICs. Population subgroups of interest include women with disabilities, children with disabilities (particularly vulnerable children with disabilities), different impairment groups, people with disabilities living in conflict settings (including conflict and post‐conflict settings), migrants with disabilities, refugees and internally displaced people with disabilities, and ethnic minorities with disabilities. All different impairment groups are eligible, including people with physical, mental, intellectual, and sensory disabilities.

#### 3.1.3 Types of interventions

There will be no restrictions on comparators/comparison groups in included studies, however to be eligible for inclusion, a study must have both an eligible intervention and an eligible outcome. As noted, eligible interventions relate to the education pillar of the WHO CBR matrix. Eligible interventions are detailed in Table 1 (although this table will be piloted before use to ensure it captures all relevant interventions).

**Table 1:** Types of interventions

Intervention domain	Intervention subcategory	Description	Setting filter: specialist or mainstream
Conditions for inclusion of people with disabilities in education	Structural interventions	Structural interventions are those targeting the context in which education takes place, such as poverty or poor resourcing of education, or community‐wide stigma and discrimination against people with disabilities	
	Learning social environment and social inclusion	Interventions aim to improve the quality and/or inclusiveness of learning social environments, and reduce stigma and discrimination	
	Accessibility of built environment and learning materials (including universal design for learning)	Interventions, including those centred on universal design, aim to improve physical accessibility of educational spaces, for instance by building ramps or developing inclusive information technology infrastructure	
	Antibullying policies and programmes	Interventions which aim to promote appreciation of diversity and prevent violence and bullying of students with disabilities, particularly young women and girls	
	Educational services development	Programmes and policy which provide for the capacity development of teachers to educate learners with a wide range of learning needs	
	Inclusive education policies	Policies are developed and implemented in mainstream and special education settings which provide for quality education for people with disabilities	
	Rehabilitation and health services, and assistive technologies	Rehabilitation and health services, and assistive technologies, are made available to learners with disabilities	
Skills for learning	Skills for formal/learning in schools	Interventions aim to equip people with disabilities with the skills necessary to pursue formal education, such as attentional capacity or time management	
	School readiness	Programmes for young children with disabilities are delivered which aim to prepare children with disabilities for participation in school on the same basis as their peers without disabilities	
	Skills for life	Youth‐ or adult‐centred learning opportunities are delivered which aim to improve the life skills and living conditions of people with disabilities, for example adult numeracy for business, or entrepreneurship development	
Attendance and enrolment	Formal enrolment	Interventions support the enrolment of people with disabilities in formal education	
	Nonformal enrolment/participation	Interventions support the enrolment of people with disabilities in various forms of nonformal education	
	Education in inclusive/mainstream settings	Opportunities are created through policy and programming for people with disabilities to meaningfully participate in mainstream education	
	School completion	Interventions support people with disabilities to complete secondary and higher education	
	Attendance	Programmes aim to support attendance at school among learners with disabilities	
Outcomes of education	Qualifications	Initiatives aim to facilitate the acquisition of relevant qualifications by people with disabilities, including high school completion certificates and training certificates	
	Education‐related quality of life	The quality of life of learners with disabilities is fostered through a variety of programmes	
	Transition to higher levels of education	Interventions support entry into post‐school opportunities on an equal basis with their peers without disabilities	

#### 3.1.4 Types of outcomes and outcome measures

Eligible outcomes, as with interventions, will largely be based on the education pillar of the CBR matrix (described in the table below). All outcomes will be considered eligible regardless of whether they are primary outcomes, or secondary outcomes. Outcomes below are organised along a causal chain, as described in the section above on intervention mechanisms (conditions for inclusive education, skills for learning, attendance and enrolment, and outcomes of education). Any adverse outcomes will be recorded under the relevant domain and flagged. The outcomes of interest include the following:

**Table:** 

Outcome domain	Outcome subcategory	Description	Setting filter: specialist or mainstream
Conditions for inclusion of people with disabilities in education	Learning social environment and social inclusion	Learning social environments are inclusive, stigma and discrimination decrease, and people with disabilities are included socially	
	Accessibility of built environment and learning materials	Classrooms and educational establishments are physically accessible to learners with disabilities, and learning materials are accessible	
	Antibullying policies and programmes implemented	Antibullying and antiviolence interventions are adequately resourced and implemented, and result in reductions in rates of bullying and violence	
	Educational services development	Teachers acquire appropriate skills to educate learners with a wide range of learning needs	
	Inclusive education policies implemented and resourced	Policies and resources are conducive to quality education for people with disabilities and ensure smooth transitions through different stages of learning	
	Rehabilitation and health services, and assistive technologies	People with disabilities have access to the necessary rehabilitation and health services and assistive technologies necessary to enable their full participation in education	
Skills for learning	Skills for formal/learning in schools	People with disabilities acquire skills which are necessary precursors to formal education	
	School readiness	Young children with disabilities are prepared for school on the same basis as their peers without disabilities	
	Skills for life	People with disabilities make use of youth or adult centred learning opportunities to improve their life skills and living conditions.	
Attendance and enrolment	Formal enrolment	People with disabilities have resources and support to enrol in quality secondary and higher education in an enabling and supportive environment and people with disabilities experience equal opportunities to participate in learning opportunities that meet their needs and respect their rights.	
	Nonformal enrolment/participation	People with disabilities participate in a variety of nonformal learning opportunities based on their needs and desires People with disabilities actively participate in early childhood developmental activities and play, either in a formal or informal environment	
	Education in inclusive/mainstream settings	People with disabilities acquire education in mainstream education facilities and	
	School completion	People with disabilities have resources and support to complete quality secondary and higher education in an enabling and supportive environment	
	Attendance	People with disabilities attend secondary and higher education	
Outcomes of education	Qualifications gained	Learners with disabilities acquire qualifications as a result of their educational participation	
	Education‐related quality of life	Learners with disabilities experience educational opportunities as positive, and as contributing to a good quality of life	
	Transition to higher levels of education	People with disabilities experience post school options on an equal basis with their peers	

#### 3.1.5 Duration of follow‐up

Any duration of follow‐up will be eligible for inclusion.

#### 3.1.6 Types of settings

All studies must be situated within a low‐ and‐middle‐income country, as defined by the World Bank (https://datahelpdesk.worldbank.org/knowledgebase/articles/906519-world-bank-country-and-lending-groups).

### 3.2 Search methods for identification of studies

The search for studies will follow two steps. First, we will conduct an electronic search of databases and sector‐specific websites. Then, after initial screening, we will examine the reference lists of all identified reviews and screen the cited studies for inclusion. We will also conduct a forward search in addition to an ancestral search. No restrictions in terms of date or format will be place on the search, but only English‐language publications will be eligible.

#### 3.2.1 Electronic searches

We propose to search all the following electronic databases:

- CINAHL
- ERIC
- Scopus
- Web of Science (Social Sciences Citation Index)
- WHO Global Health Index
- MEDLINE(R)
- Embase Classic+Embase
- PsycINFO
- CAB Global Health.

MEDLINE, Embase, PsychINFO, and CAB Global Health will be searched through OVID, and ERIC and CINAHL through Ebsco. PubMED will be searched through NCBI. We will tailor the search strategy for each of the databases. However, the main search strategy will include the following population, study design and location terms:

**POPULATION**: (disable* or disabilit* or handicapped) **OR** (physical* or intellectual* or learning or psychiatric* or sensory or motor or neuromotor or cognitive or mental* or developmental or communication or learning) **OR** (cognitive* or learning or mobility or sensory or visual* or vision or sight or hearing or physical* or mental* or intellectual*) adj2 (impair* or disabilit* or disabl* or handicap*) **OR** (communication or language or speech or learning) adj5 (disorder*) **OR** (depression or depressive or anxiety or psychiat* or well‐being or quality of life or self‐esteem or self perception) adj2 (impair* or disabilit* or disabl* or handicap*) **OR** mental health **OR** (schizophreni* or psychos* or psychotic or schizoaffective or schizophreniform or dementia* or alzheimer*) adj2 (impair* or disabilit* or disabl* or handicap*) **OR** (mental* or emotional* or psychiatric or neurologic*) adj2 (disorder* or ill or illness*) **OR** (autis* or dyslexi* or Down* syndrome or mongolism or trisomy 21) **OR** (intellectual* or educational* or mental* or psychological* or developmental) adj5 (impair* or retard* or deficien* or disable* or disabili* or handicap* or ill*) **OR** (hearing or acoustic or ear*) adj5 (loss* or impair* or deficien* or disable* or disabili* or handicap* or deaf*) **OR** (visual* or vision or eye* or ocular) adj5 (loss* or impair* or deficien* or disable* or disabili* or handicap* or blind*) **OR** (cerebral pals* or spina bifida or muscular dystroph* or arthriti* or osteogenesis imperfecta or musculoskeletal abnormalit* or musculo‐skeletal abnormalit* or muscular abnormalit* or skeletal abnormalit* or limb abnormalit* or brain injur* or amput* or clubfoot or polio* or paraplegi* or paralys* or paralyz* or hemiplegi* or stroke* or cerebrovascular accident*) adj2 (impair* or disabilit* or disabl* or handicap*) **OR** (physical* adj5 (impair* or deficien* or disable* or disabili* or handicap*) **OR** people with disabilities/or children with disabilities/or people with mental disabilities/or people with physical disabilities/**OR** abnormalities/or exp congenital abnormalities/or exp deformities/or exp disabilities/or exp malformations/**OR** exp mental disorders/or exp mental health/or learning disabilities/or paralysis/or paraparesis/or paraplegia/or poliomyelitis/or hearing impairment/or deafness/or people with hearing impairment/or vision disorders/or blindness/or people with visual impairment/.

**STUDY DESIGN**: (systematic* or synthes*) adj3 (research or evaluation* or finding* or thematic* or report or descriptive or explanatory or narrative or meta* or review* or data or literature or studies or evidence or map or quantitative or study or studies or paper or impact or impacts or effect* or compar*) **OR** ("meta regression" or "meta synth*" or "meta‐synth*" or "meta analy*" or "metaanaly*" or "meta‐analy*" or "metanaly*" or "metaregression" or "metaregression" or "methodologic* overview" or "pool* analys*" or "pool* data" or "quantitative* overview" or "research integration") **OR** (review adj3 (effectiveness or effects or systemat* or synth* or integrat* or map* or methodologic* or quantitative or evidence or literature)) **OR** ("meta ethnograph*" or "meta synthesis" or (synthesis and ("qualitative literature" or "qualitative research")) or "critical interpretive synthesis" or ("systematic review" and ("qualitative research" or "qualitative literature" or "qualitative stud*")) or "thematic synthesis" or "framework synthesis" or "realist review" or "realist synthesis" or "qualitative systematic review*" or "qualitative evidence synthes*" or (("quality assessment" or "critical appraisal" or "literature search*") and ("qualitative research" or "qualitative literature" or "qualitative stud*")) or (Noblit and Hare) or "meta narrative*" or "narrative synthesis") **OR** meta‐analysis/or evaluation studies/or qualitative research/or systematic review/**OR** controlled clinical trial/or randomised controlled trial/or equivalence trial/or pragmatic clinical trial/or case‐control studies/or retrospective studies/or cohort studies/or follow‐up studies/or longitudinal studies/or prospective studies/or epidemiologic methods/or epidemiologic studies/or controlled before‐after studies/or cross‐sectional studies/or interrupted time series analysis/or control groups/or cross‐over studies/or double‐blind method/or matched‐pair analysis/or meta‐analysis as topic/or random allocation/or single‐blind method/or "retraction of publication"/or case reports/**OR** (random or placebo or single blind or double blind or triple blind or cohort or ((case or cohort or follow up or follow‐up) adj2 (control or series or report or study or studies)) or retrospective or (observ adj3 (study or studies))).

**LOCATION**: Developing Countries **OR** Africa/or Asia/or Caribbean/or West Indies/or Middle East/or South America/or Latin America/or Central America/**OR** (Africa or Asia or Caribbean or West Indies or Middle East or South America or Latin America or Central America) **OR** ((developing or less* developed or under developed or underdeveloped or middle income or low* income or underserved or under served or deprived or poor*) adj (countr* or nation? or population? or world or state*)) **OR** ((developing or less* developed or under developed or underdeveloped or middle income or low* income) adj (economy or economies)) **OR** (low* adj (gdp or gnp or gross domestic or gross national)) **OR** (low adj3 middle adj3 countr*) **OR** (lmic or lmics or third world or lami countr*) **OR** transitional countr*.

#### 3.2.2 Searching other resources

As noted, we will search the reference lists of identified recent papers and reviews. However, we will also take the necessary steps to ensure that we cover the unpublished literature, so as to minimise the risk of publication bias in our review. To this end, we will search the following websites and databases using a tailored keyword search for unpublished grey literature:

- International Labour Organisation
- Department For International Development (including Research for Development [R4D])
- United Nations Educational, Scientific and Cultural Organisation
- World Health Organization
- Disability Programme of the United Nations Economic and Social Commission for Asia and the Pacific
- United States Agency for International Development
- Dissertation Abstracts, Conference Proceedings and Open Grey
- Humanity and Inclusion http://www.hi-us.org/publications
- CBM https://www.cbm.org/Publications-252011.php
- Plan International https://plan-international.org/publications

### 3.3 Data collection and analysis

#### 3.3.1 Description of methods used in primary research

We will use an online software program, EppiReviewer (https://eppi.ioe.ac.uk/), for bibliographic management, screening, coding and data synthesis. The screening checklist will be reviewed by H.K. and H.W. Eligibility will be assessed using a predesigned form based on the inclusion criteria (see Annex 1). We intend to pilot all coding sheets with at least five studies before use. The forms allow for coding of multiple intervention domains and multiple outcomes domains. All changes to these criteria will be reported in the final SR. Articles excluded at this stage will be reported in a flow chart with reasons for exclusion. The entire screening process will be reported using a PRISMA flow chart.

#### 3.3.2 Criteria for determination of independent findings

Where there are multiple publications reporting on the same study, we will examine the publications as a single study.

#### 3.3.3 Selection of studies

We will screen all unique references from our search title and abstract, with two independent reviewers determining relevance. If any disagreement arises, it will be resolved by third independent reviewer. A similar process will be followed for full texts: the full text of article which appear relevant on title and abstract will be screened independently by two independent reviewers, with disagreement resolved by third reviewer. These disagreements will be discussed with H.K. We will report interrater reliability for study identification.

#### 3.3.4 Data extraction and management

Two independent data collectors will code the included studies. They will extract data from the studies according to a coding sheet (Annex 2), and a third data collector will check the results of this process. Studies will be coded by intervention, outcomes and a range of filters such as age of target population, and types of disabilities covered. Where appropriate and possible, we will also extract the following methodological and quantitative data:

- Study design
- Analysis method
- Type of comparison (if relevant)
- External validity
- Outcome descriptive information
- Sample size in each intervention group
- Outcomes means and standard deviations
- Test statistics (e.g., *t* test, *F* test, *p* values, 95% confidence intervals)
- Information on intervention design.

As noted, where systematic reviews are discovered by the search, their reference list of primary studies will be assessed for eligibility. As such, the proposed systematic review will not include summarised findings of the systematic reviews to avoid duplication.

#### 3.3.5 Quality assessment and assessment of risk of bias in included studies

Table 2 presents the tool which we will use to assess confidence in study findings. This tool,^1^ which the authors are using across a range of disability intervention systematic reviews, contains six criteria:

1. **Study design**: We want to see that potential confounders have been considered. Impact evaluations, in which we are interested, must have either a well‐designed control group, preferably based on random assignment, or an estimation technique which controls for confounding and the associated possibility of selection bias.
2. **Masking**: Masking, or blinding, is only relevant in RCTs. This procedure helps to limit the biases which can occur if study participants, data collectors or data analysts are aware of the assignment condition of individual participants.
3. **Presence of a power calculation**: many studies may be underpowered, but it is difficult to assess without the inclusion in the study of a power calculation.
4. **Attrition**: This can be a major source of bias in studies, especially if there is differential attrition between the treatment and comparison group so that the two may no longer be balanced in pre‐intervention characteristics. The US Institute of Education Sciences What Works Clearing House has developed standards for acceptable levels of attrition, in aggregate and the differential, which we will apply.^2^
5. **Clear definition of disability**: For a study to be useful the study population must be clear, which means that the type and degree of disability should be clearly defined, preferably with reference to a widely used international standard.
6. **Clear definition of outcome measures**: To aid interpretation and reliability of findings and comparability with other studies, outcome measures must be clearly defined. Studies should state the outcomes being used with a definition and the basis on which they are measured, preferably with reference to a widely used international standard.
7. **Baseline balance**: This shows that the treatment and comparison groups are the same at baseline. Lack of balance between groups at baseline can bias the results.

**Table 2:** Study quality assessment criteria

	Criterion	Low	Medium	High
1	Study design (potential confounders considered)	Before versus after. Naïve matching	IV, RDD, PSM, double difference	RCT, natural experiment
2	Blinding (RCTs only)	No mention of blinding	Blinding for analysis.	Blinding of data collection (where feasible). Blinding for analysis.
3	Losses to follow up are presented and acceptable	Attrition not reported, OR falls well outside WWC acceptable combined levels*	Overall and differential attrition close to WWC combined levels*	Overall and differential attrition within WWC combined levels*
4	Disability/impairment measure is clearly defined and reliable	No definition OR overall attrition >50%	Unclear definition OR Single question item only (e.g., are you disabled)	Clear definition, for example, Washington Group questions, detailed measure of impairment
5	Outcome measures are clearly defined and reliable	No definition	Unclear definition	Clear definition using existing measure where possible
6	Baseline balance (NA for before vs. after)	No baseline balance test (except RCT) OR reported and significant differences on more than five measures. PSM without establishing common support.	Baseline balance test, imbalance on 5 or fewer measures	RCT, RDD
7	Overall confidence in study findings	Low on any item	Medium or high confidence on all items	RCT with high confidence on all items

Confidence in study findings will be rated as high, medium or low, for each of the criteria, applying the standards as shown in Table 1. Overall confidence in study findings will be the lowest rating a study achieves across the criteria (using the weakest link in the chain principle).

Where a study reports outcomes at more than one point in time it is possible that the study quality varies between those two points for two of the criteria: (1) an RCT may no longer be so if it used a waitlist or pipeline design so the control group has received the treatment (item 1), (2) there may be greater attrition rates at the later point in time. In applying the tool, an assessment will be made for the earliest and latest outcome measures for items 1 and 4, and confidence in study findings will be assessed separately for the two points in time.

#### 3.3.6 Measures of treatment effect

Any measure of treatment effect will be eligible for inclusion and analysis in this review, should a meta‐analysis be performed. These include both ratio measures (e.g., risk ratio, odds ratio) and difference measures (e.g., mean difference, risk difference). Where measures of treatment effect are reported, we will calculate effect sizes, which is a standardised expression of the magnitude or strength of the relationship of interest (Borenstein et al., 2009a; Valentine & Cooper, 2003).

Our treatment of quantitative studies in general, including calculations of measures of effect, is guided by the Campbell protocol of Waddington et al. (2018), as it provides comprehensive guidance in terms of calculating effect sizes where heterogeneous studies are expected.

For continuous outcomes comparing group means in an intervention and control group, we will calculate the standardised mean difference (SMDs), or Cohen's *d*, its variance and standard error using formulae provided in Borenstein et al. (2009b) and as recommended by Waddington et al. (2018). Waddington et al. (2018) recommend Cohen's *d* be adjusted using Hedges' method (below) where sample sizes are small, to avoid bias (Ellis, 2010):

$$g≅d(1-\frac{3}{4\left(n_{1}+n_{2}\right)-9}).$$

Formulas for effect size calculations will be used depending on data provided in included studies. If the study does not report the pooled standard deviation, it will be calculated.

Where the intervention is expected to change the standard deviation of the outcome variable, we will use the standard deviation of the control group only.

We will analyse studies reporting means ($\underline{X}$) and SD for treatment and control or comparison groups at baseline (*p*) and follow up (*p* + 1); studies reporting mean differences $\left(\Delta \underline{X}\right)$ between treatment and control and SD at follow up (*p* + 1); and studies reporting mean differences between treatment and control, SE and sample size (*n*), using the appropriate formulae described in Waddington et al. (2018).

Where included studies contain regression analyses, we will use the regression coefficient and the pooled standard deviation of the outcome or the standard errors to calculate effect sizes^3^ (Waddington et al., 2018). We will calculate the *t* statistic (*t*) by dividing the coefficient by the standard error, and in cases in which significance levels are reported rather than *t*, we will impute *t* according to the guidelines set out in Waddington et al. (2018).

For studies reporting proportions (including odds or rate ratios), we will also calculate effect size (using the Cox‐transformed log odds ratio effect size for proportions of individuals and the standardised proportion difference effect size for proportions of events or time). Where we identify several papers that report on the same study, we will use effect sizes from the most recent publication. However, where different publications report on the same intervention, but refer to different sub‐samples of the overall study population, we will treat each sample as an independent sample.

#### 3.3.7 Unit of analysis issues

Based on a Rapid Evidence Assessment on the topic of education for people with disabilities which preceded this proposed SR, we anticipate that most interventions, if using randomisation, will utilise individual randomisation. However, we will include cluster‐randomised studies and crossover studies. We will firstly assess whether the study authors have adequately accounted for clustering or correlation in the analysis reported in the paper. If they have not, we will either reanalyse the results in line with the recommendations outlined in the Cochrane Handbook for clustered designs (Chapter 23), or we will include only the first time point (for crossover studies) in our meta‐analysis. Where more than two intervention groups are reported, we will omit groups that are not relevant to the comparison being made and combine multiple groups that are eligible as either the intervention or control/comparator, to allow for a single pair‐wise comparison.

#### 3.3.8 Dealing with missing data

In case of missing information, the author(s) of the original study will be contacted. We will document correspondence with study authors.

#### 3.3.9 Assessment of heterogeneity

If possible and appropriate, heterogeneity analyses will be conducted for outcomes. We will also calculate standardised effect sizes where possible. In light of the fact that multiple effect sizes may be attributable to sampling error, a random effects model and the associated inverse variance weight at the 95% confidence level will be used for all analyses. The random effects model provides for an assumption of population variation from which the sample is drawn and calculates the impact of the effect size by estimating the parameters of that population. Additionally, the random effects model provides the opportunity to account for studies not included in the current data set thus allowing for generalisation beyond the present study.

#### 3.3.10 Assessment of reporting biases

Assessment of reporting biases is covered under the section above “Quality assessment and assessment of risk of bias in included studies”.

#### 3.3.11 Data synthesis

Coding will include: (1) extraction of basic study characteristics, (2) a narrative summary of procedures and findings (including recording of iatrogenic effects), (3) a summary of findings/results table, and (4) a quality assessment. All data will be extracted from the studies according to an extraction table (see Annex 2). This coding will be conducted by two coders, with comparison and discussion with a third person (H.K. or H.W.) to resolve any disagreements.

As noted under “Assessment of Heterogeneity”, we will code effect sizes. However, if there is too much variation in the reporting of outcomes (such that they are not comparable), as well as in effect sizes, we will not perform a meta‐analysis.

If, however, we find that it is possible to conduct a meta‐analysis, we will do so. Given the expected heterogeneity, it is hard to predict how meta‐analysis might be used in the review. Nonetheless, following Waddington et al. (2018), we will only combine studies using meta‐analysis when we identify two or more effect sizes using a similar outcome construct and where the comparison group is similar across the two studies. If possible, we will conduct separate analyses for the major outcome groups:

- Conditions for inclusion of people with disabilities in education
- Skills for learning
- Attendance and enrolment
- Outcomes of education.

We do not plan a subgroup analysis. However, in the final narrative, attention will be paid to describing findings according to suboutcome, that is, by: learning social environment and social inclusion; accessibility of built environment and learning materials; anti‐bullying policies and programmes implemented; educational services development; inclusive education policies implemented and resourced; skills for formal/learning in schools; school readiness; skills for life; formal enrolment; nonformal enrolment/participation; education in inclusive/mainstream settings; school completion; attendance; qualifications gained; education‐related quality of life; and transition to higher levels of education. In each of these suboutcome narratives, the main themes and findings will be described, and note will be made of the strength of the evidence, any evidence gaps, and confidence in the evidence.

#### 3.3.12 Subgroup analysis and investigation of heterogeneity

If possible and appropriate, heterogeneity analyses will be conducted for outcomes. Because multiple effect sizes may be attributable to sampling error, a random effects model and the associated inverse variance weight at the 95% confidence level will be used for all analyses. The random effects model provides for an assumption of population variation from which the sample is drawn and calculates the impact of the effect size by estimating the parameters of that population. Additionally, the random effects model provides the opportunity to account for studies not included in the current data set thus allowing for generalisation beyond the present study.

Even if we cannot conduct a meta‐analysis, we are interested in certain specific populations of people with disabilities, including women, children (particularly vulnerable children, e.g., those in care), different impairment groups, conflict (conflict and postconflict settings), migrants/refugees/internally displaced people, and ethnic minority groups. For papers addressing these issues, we will extract effect sizes and if data allows, disaggregate outcome findings by group. However, our expectation is that we will instead be able to provide a narrative description of any apparent notable characteristics of papers addressing these groups, but these findings will be descriptive and tentative.

#### 3.3.13 Treatment of qualitative research

We will not include qualitative research in this systematic review.

## EXTERNAL SOURCES

This systematic review is supported by the UK Department of International Development (DFID) under its support for the Centre for Excellence for Development Impact and Learning (CEDIL) and the Programme for Evidence to iNform Disability Action (PENDA).

## Supporting information

Supporting information.

## Footnotes

1 Thanks also to Hugh Waddington (3ie and Campbell IDCG) for suggestions used in developing the tool.

2 See Table 1 https://homvee.acf.hhs.gov/HomVEE-Attrition-White_Paper-7-2015.pdf.

3 If the authors only report confidence intervals, we will calculate the standard error from the confidence intervals.
