# Supplementary material for: PROTOCOL: Effectiveness of interventions for improving educational outcomes for people with disabilities in low‐ and middle‐income countries: A systematic review
Source: Campbell Syst Rev. 2021 Oct 23;17(4):e1197. doi: 10.1002/cl2.1197 (PMC8988635; doi:10.1002/cl2.1197)
Supplement: Supplementary file 1 — Supporting information. [file CL2-17-e1197-s001.docx]

# Appendices

## 1 Annex 1

Annex 1

### The screening checklist will include the following:

1. Does the study include a relevant intervention AND a relevant outcome (see intervention nad outcome tables)?

2. Is the study conducted with people with disabilities living in low- and middle-income countries?

3. Is the study one in which participants are randomly assigned or quasi-randomly assigned, or where non-random assignment has been done, but participants have been matched on pre-tests and/or relevant demographic characteristics or statistical methods have been used to control for differences between groups; or where the design attempts to detect whether the intervention has had an effect significantly greater than any underlying trend over time, using observations at multiple time points before and after the intervention (interrupted time-series design); or where participants receiving an intervention are compared with a similar group from the past who did not (i.e., a historically controlled study); or where observations are made on a group of individuals before and after an intervention, but with no control group (single-group before-and-after study).

## 2 Annex 2

| **Code intervention:** | | | |
| --- | --- | --- | --- |
| Intervention Domain | Intervention sub-category | Description | Setting Filter: Specialist or mainstream |
| Conditions for inclusion of people with disabilities in education | Learning social environment and social inclusion | Interventions aim to improve the quality and/or inclusiveness of learning social environments, and reduce stigma and discrimination |  |
|  | Accessibility of built environment and learning materials | Interventions, including those centered on universal design, aim to improve physical accessibility of educational spaces, for instance by building ramps or developing inclusive information technology infrastructure |  |
|  | Anti-bullying policies and programmes | Interventions which aim to promote appreciation of diversity and prevent violence and bullying of students with disabilities, particularly young women and girls |  |
|  | Educational services development | Programmes and policy which provide for the capacity development of teachers to educate learners with a wide range of learning needs |  |
|  | Inclusive education policies | Policies are developed and implemented in mainstream and special education settings which provide for quality education for people with disabilities |  |
|  | Rehabilitation and health services, and assistive technologies | Rehabilitation and health services, and assistive technologies, are made available to learners with disabilities |  |
| Skills for learning | Skills for formal/learning in schools | Interventions aim to equip people with disabilities with the skills necessary to pursue formal education, such as attentional capacity or time management |  |
|  | School readiness | Programmes for young children with disabilities are delivered which aim to prepare children with disabilities for participation in school on the same basis as their peers without disabilities |  |
|  | Skills for life | Youth- or adult- centered learning opportunities are delivered which aim to improve the life skills and living conditions of people with disabilities, for example adult numeracy for business, or entrepreneurship development |  |
| Attendance and enrollment | Formal enrollment | Interventions support the enrollment of people with disabilities in formal education |  |
|  | Nonformal enrollment/participation | Interventions support the enrollment of people with disabilities in various forms of nonformal education |  |
|  | Education in inclusive/mainstream settings | Opportunities are created through policy and programming for people with disabilities to meaningfully participate in mainstream education |  |
|  | School completion | Interventions support people with disabilities to complete secondary and higher education |  |
|  | Attendance | Programmes aim to support attendance at school among learners with disabilities |  |
| Outcomes of education | Qualifications | Initiatives aim to facilitate the acquisition of relevant qualifications by people with disabilities, including high school completion certificates and training certificates |  |
|  | Education-related quality of life | The quality of life of learners with disabilities is fostered through a variety of programmes |  |
|  | Transition to higher levels of education | Interventions support entry into post-school opportunities on an equal basis with their peers without disabilities |  |
| Code outcome: | | | |
| Outcome Domain | Outcome sub-category | Description | Setting Filter: Specialist or mainstream |
| Conditions for inclusion of people with disabilities in education | Learning social environment and social inclusion | Learning social environments are inclusive, stigma and discrimination decrease, and people with disabilities are included socially |  |
|  | Accessibility of built environment and learning materials | Classrooms and educational establishments are physically accessible to learners with disabilities, and learning materials are accessible |  |
|  | Anti-bullying policies and programmes implemented | Anti-bullying and anti-violence interventions are adequately resourced and implemented, and result in reductions in rates of bullying and violence |  |
|  | Educational services development | Teachers acquire appropriate skills to educate learners with a wide range of learning needs |  |
|  | Inclusive education policies implemented and resourced | Policies and resources are conducive to quality education for people with disabilities and ensure smooth transitions through different stages of learning |  |
|  | Rehabilitation and health services, and assistive technologies | People with disabilities have access to the necessary rehabilitation and health services and assistive technologies necessary to enable their full participation in education |  |
| Skills for learning | Skills for formal/learning in schools | People with disabilities acquire skills which are necessary precursors to formal education |  |
|  | School readiness | Young children with disabilities are prepared for school on the same basis as their peers without disabilities |  |
|  | Skills for life | People with disabilities make use of youth or adult centered learning opportunities to improve their life skills and living conditions. |  |
| Attendance and enrollment | Formal enrollment | People with disabilities have resources and support to enroll in quality secondary and higher education in an enabling and supportive environment and people with disabilities experience equal opportunities to participate in learning opportunities that meet their needs and respect their rights. |  |
|  | Nonformal enrollment/participation | People with disabilities participate in a variety of nonformal learning opportunities based on their needs and desires People with disabilities actively participate in early childhood developmental activities and play, either in a formal or informal environment |  |
|  | Education in inclusive/mainstream settings | People with disabilities acquire education in mainstream education facilities and |  |
|  | School completion | People with disabilities have resources and support to complete quality secondary and higher education in an enabling and supportive environment |  |
|  | Attendance | People with disabilities attend secondary and higher education |  |
| Outcomes of education | Qualifications gained | Learners with disabilities acquire qualifications as a result of their educational participation |  |
|  | Education-related quality of life | Learners with disabilities experience educational opportunities as positive, and as contributing to a good quality of life |  |
|  | Transition to higher levels of education | People with disabilities experience post school options on an equal basis with their peers |  |
| Code participant characteristics: | | | |
| **Participant target group** | | | |
| *Who was the main recipient of the treatment (Target group?)* |  |  | |
| People with disability | Child (0-17.9 years) |  | |
|  | Adults |  | |
|  | Elderly |  | |
|  |  |  | |
| Family member/caregiver |  |  | |
| Service provider/professional/teachers |  |  | |
| Community member |  |  | |
| Other (specify) |  |  | |
|  |  |  | |
| **Gender of target group** | | | |
| Male |  |  | |
|  |  |  | |
| Female |  |  | |
| Both |  |  | |
| Not reported |  |  | |
|  |  |  | |
| **Participants SES** | | | |
| *What was the SES of treatment group?* |  |  | |
| Low |  |  | |
| Middle |  |  | |
| High |  |  | |
| Mixed |  |  | |
| Can't tell/not reported |  |  | |
|  |  |  | |
| **Type of disability** | | | |
| *What was the predominant disability/health condition of the treatment group?* | | | |
| Hearing |  |  | |
| Physical |  |  | |
| Visual |  |  | |
| Intellectual/learning and developmental/behavioural |  |  | |
| Mental | e.g. schizophrenia |  | |
| Can't tell/not reported |  |  | |
|  |  |  | |
| **World Bank region** | | | |
| East Asia and Pacific |  |  | |
| Europe and Central Asia |  |  | |
| Latin America and Caribbean |  |  | |
| Middle East and North Africa |  |  | |
| Sub-Saharan Africa |  |  | |
| South Asia |  |  | |
|  |  |  | |
| **Country of study (specify)** |  |  | |
|  |  |  | |
| **Geographical setting of the intervention** | | | |
| Urban |  |  | |
| Rural |  |  | |
| Mixed |  |  | |
| Can't tell/not reported |  |  | |
| others (specify) |  |  | |
|  |  |  | |
| **Study design** | | | |
| RCT |  |  | |
| Controlled before and after |  |  | |
| Uncontrolled before and after |  |  | |
| RDD |  |  | |
| ITS |  |  | |
| Matched designs |  |  | |
| Others (specify) |  |  | |
|  |  |  | |
| **Subject assignment?** | | | |
| Individual random |  |  | |
| Whole group random |  |  | |
| Individual matched‐random |  |  | |
| Non‐matched and non‐random |  |  | |
| Cannot tell/Not reported |  |  | |
| Other (Specify): |  |  | |
